# Visual and Plasmon Resonance Absorption Sensor for Adenosine Triphosphate Based on the High Affinity between Phosphate and Zr(IV)

**DOI:** 10.3390/s16101674

**Published:** 2016-10-12

**Authors:** Wenjing Qi, Zhongyuan Liu, Wei Zhang, Mohamed Ibrahim Halawa, Guobao Xu

**Affiliations:** 1 State Key Laboratory of Electroanalytical Chemistry, Changchun Institute of Applied Chemistry, Chinese Academy of Sciences, Changchun 130022, China; zhongyuanliu@ciac.ac.cn (Z.L.); mirandazhang@ciac.ac.cn (W.Z.); abohalawa@ciac.ac.cn (M.I.H.); 2 Chongqing Key Laboratory of Green Synthesis and Applications, College of Chemistry, Chongqing Normal University, Chongqing 401331, China; 3 Chinese Academy of Sciences, University of Chinese Academy of Sciences, No. 19A Yuquanlu, Beijing 100049, China

**Keywords:** gold nanoparticles, adenosine triphosphate, plasmon resonance absorption, sensor

## Abstract

Zr(IV) can form phosphate and Zr(IV) (–PO_3_^2−^–Zr^4+^–) complex owing to the high affinity between Zr(IV) with phosphate. Zr(IV) can induce the aggregation of gold nanoparticles (AuNPs), while adenosine triphosphate(ATP) can prevent Zr(IV)-induced aggregation of AuNPs. Herein, a visual and plasmon resonance absorption (PRA)sensor for ATP have been developed using AuNPs based on the high affinity between Zr(IV)with ATP. AuNPs get aggregated in the presence of certain concentrations of Zr(IV). After the addition of ATP, ATP reacts with Zr(IV) and prevents AuNPs from aggregation, enabling the detection of ATP. Because of the fast interaction of ATP with Zr(IV), ATP can be detected with a detection limit of 0.5 μM within 2 min by the naked eye. Moreover, ATP can be detected by the PRA technique with higher sensitivity. The *A*_520nm_/*A*_650nm_ values in PRA spectra increase linearly with the concentrations of ATP from 0.1 μM to 15 μM (r = 0.9945) with a detection limit of 28 nM. The proposed visual and PRA sensor exhibit good selectivity against adenosine, adenosine monophosphate, guanosine triphosphate, cytidine triphosphate and uridine triphosphate. The recoveries for the analysis of ATP in synthetic samples range from 95.3% to 102.0%. Therefore, the proposed novel sensor for ATP is promising for real-time or on-site detection of ATP.

## 1. Introduction

Adenosine triphosphate (ATP), as cellular energy currency in living cells, is one of the most important small-molecule signaling agents [1,2,3,4,5]. It is an indicator for cell viability and cell injury and plays an important role in many biological processes such as the regulation of cellular metabolism and biochemical pathways in cell physiology. Intracellular ATP levels are related to many diseases, such as hypoxia, hypoglycemia, ischemia, Parkinson’s disease, and some tumors [6,7,8]. Moreover, the measurements of the concentrations of ATP in real samples are required for bacteria detection in food and agricultural chemistry to prevent food-borne illnesses and in the health sciences [4]. Therefore, exploring methods for sensitive and selective determination of ATP is of great importance in biochemical analysis and clinical diagnoses. Up to now, different methods have been used for the detection of ATP, such as electrochemiluminescence [9,10,11,12,13], electrochemistry [5,14,15,16,17,18], fluorescence [19,20,21,22], and localized surface plasmon resonance [1]. Most of these strategies need the participation of enzymes or aptamers. They usually need half an hour or even longer for the reaction between ATP and its aptamer or enzymatic reaction. Meanwhile, enzymes or aptamers as biochemical reagents need some special storage methods. Both of them limit the fast response in the analysis of ATP. To some extent, some sensitive and selective sensors for ATP without the need of enzymes or aptamers are potentially available in real-time or on-site detection.

Owing to the different color changes and plasmon resonance absorption (PRA) properties between dispersed and aggregated AuNPs, AuNPs (mostly 13 nm AuNPs synthesized by trisodium citrate) have been widely employed in developing visual and PRA chemical and biological sensors for metal ions (such as Hg(II), As(III), Cu(II)), biochemical small molecules (such as ATP, dopamine), DNA and proteins [1,23,24,25,26,27,28,29,30,31,32,33,34,35,36]. The reported visual and PRA sensors for ATP usually need aptamers. The main principle of ATP sensors relies on the changes that aptamers change into a G-quartet folding structure after aptamers react with ATP. Since it usually takes a long incubation time (usually half an hour) for the formation of the aptamer and ATP complex, the ATP sensors free from aptamers with a fast response within several seconds would be good alternatives. Using unmodified AuNPs (13 nm), Deng developed a simple and fast method for ATP detection free from aptamers based on the high adsorption affinity of ATP on the surface of AuNPs and Cu^2+^-induced cross-linking aggregation [32]. In their report, Cu^2+^ forms a dimeric [Cu_2_(ATP)]_2_ complex with ATP to achieve aggregation. Liu reported a colorimetric method for ATP detection free from aptamers using unmodified AuNPs (13 nm) [31]. It is a responsive disassembly of AuNP aggregates triggered by the competitive adsorption for lighting up the colorimetric sensing. In the presence of melamine, AuNPs can form aggregates. However, since the ATP molecule has higher adsorption affinity than that of the melamine on the surface of AuNPs, AuNPs get dispersed on the basis of the competitive adsorption after the addition of ATP, enabling the determination of ATP. 

**Scheme 1:**
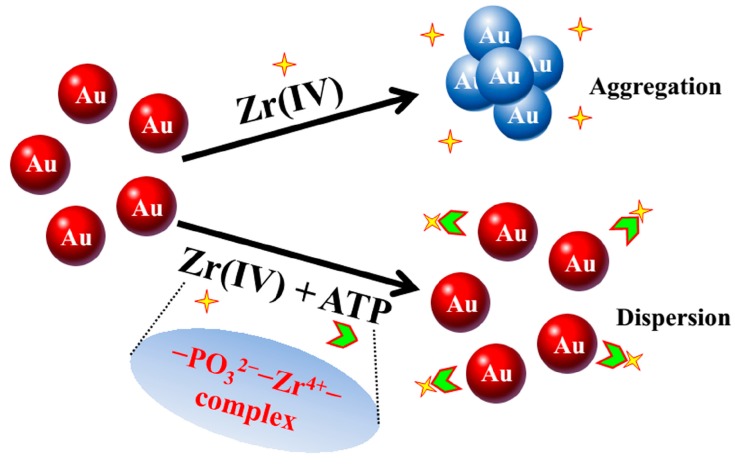
Schematic illustration of visual and PRA sensor for ATP based on the high affinity between phosphate and Zr(IV).

Zr(IV) can form phosphate and Zr(IV) (–PO_3_^2−^–Zr^4+^–) owing to the high affinity between the Zr(IV) and phosphate. This special property between Zr(IV) and phosphate has been mostly utilized in the immobilization of phosphopeptides and phosphate-functionalized DNA or as linkers for peptide and enzyme conjugates [37,38,39]. Inspired by these reports, we utilize the high affinity of Zr(IV) and phosphate in developing a new visual sensor for ATP without the need of aptamers in this study since ATP has phosphate groups. As shown in Scheme 1, Zr(IV) can make well-dispersed AuNP aggregates. ATP with a phosphate group has high affinity with Zr(IV) and can form phosphate and Zr(IV) (–PO_3_^2−^–Zr^4+^–) with Zr(IV). As a result, the novel visual and PRA sensor for ATP is designed using AuNPs on the basis of dispersed and aggregated states of AuNPs. It is the first investigation for ATP detection using the high affinity between phosphate and Zr(IV). Compared with other reports, the proposed sensor for ATP using AuNPs is sensitive, simple and fast within 2 min. More importantly, the proposed sensor only needs the participation of inorganic ions Zr(IV) without the need of aptamers, which saves the incubating time of ATP with its aptamer and cost. It is available for real-time or on-site detection of ATP.

## 2. Materials and Methods

### 2.1. Materials and Reagents

ATP disodium salt hydrate, adenosine, adenosine diphosphate (ADP), adenosine monophosphate (AMP), guanosine triphosphate (GTP), cytidine triphosphate (CTP) and uridinetriphosphate (UTP) were purchased from Sigma-Aldrich. Zirconium acetate (C_2_H_4_O_4_Zr) was purchased from Aladdin (Beijing, China). Tetrachloroaurate (III) tetrahydrate (HAuCl_4_·4H_2_O) and trisodium citrate (Na_3_C_6_H_5_O_7_·2H_2_O) were purchased from Sinopharm Chemical Reagent Co., Ltd. (Shanghai, China). Other chemicals were of analytical-reagent grade and used as received. All solutions were prepared with doubly distilled water.

### 2.2. Apparatus

Plasmon resonance absorption (PRA) spectra were measured using a UNICO UV/VIS2802PC spectrophotometer. Transmission electron microscopy (TEM) images were obtained by a Hitachi H600 transmission electron microscope operated at 100 kV. AuNPs samples for TEM measurements were prepared by placing a drop of colloidal solution on carbon-coated copper grid and then dried at room temperature. A WH-3 miniature vortex mixed instrument (Shanghai Hu Xi Analysis Instrument Factory Co., Ltd, Shanghai, China) was used to mix the samples thoroughly.

### 2.3. The Preparation of AuNPs

AuNPs were prepared similarly with our previous reports [40,41] by reducing HAuCl_4_ with citrate sodium, which acts as both reducing agent and stabilizer. Doubly distilled water (48 mL) and HAuCl_4_ (1%, 2 mL) with final concentration of 1 mM were firstly added in a flask. Then the mixture was stirred and heated to be boiling. Then trisodium citrate (5%, 1 mL) was added to the flask by keeping boiling. The solution changes from pale yellow to deep red within 3 min. After boiling for another 5 min, the solution was cooled to room temperature under magnetic stirring. At last, AuNPs was stored in a refrigerator (4 °C). The approximate concentrations of AuNPs (about 13 nm) are calculated according to previous reports [40,41].

### 2.4. General Procedures of ATP Detection

Tris-HCl buffer solutions (pH 7.4, 100 μL), Zr(IV) (10 μM) and different concentrations of ATP were firstly pipetted into a 1.0mL plastic tube. The mixture was vortex-mixed and kept at room temperature for 10 min. Then AuNPs (100 μL), appropriate volume of water and NaCl solutions with the final concentration of 30 mM (to control the medium ionic strength of the system) were added. The whole mixture was vortex-mixed thoroughly, kept at room temperature for another 2 min and transferred for PRA spectra measurements.

### 2.5. ATP Detection in Synthetic Mixture

ATP (1, 5 and 10 μM) was respectively added to synthetic mixtures containing adenosine, ADP, AMP, GTP, CTP and UTP. Then tris-HCl buffer solutions (pH 7.4, 100 μL) and Zr(IV) (10 μM) were added. Then AuNPs were added and NaCl (30 mM) was added at last. The whole solutions were vortex-mixed thoroughly, kept at room temperature for another 2 min and transferred for PRA spectra measurements.

## 3. Results and Discussions

### 3.1. State of AuNPswith ATP in the Presence of Zr(IV)

**Figure 1:**
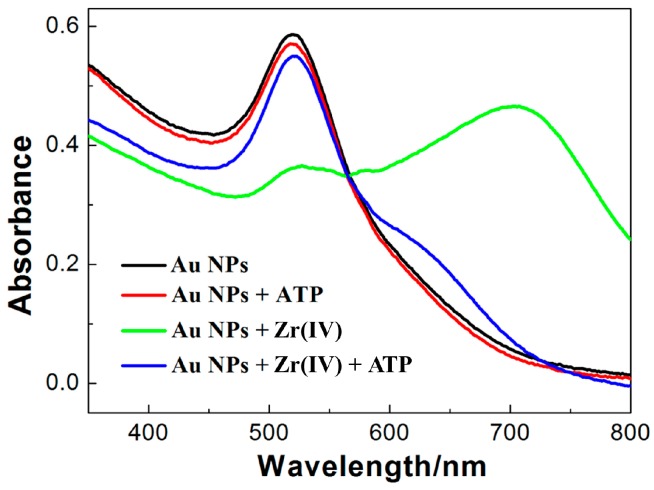
Absorption spectra of AuNPs in different solutions. Black curve represents PRA spectra of the as-prepared AuNPs solution (no NaCl); Red curve represents PRA spectra of AuNPs with ATP (30 mM NaCl); Green curve represents PRA spectra of AuNPs with Zr(IV) (30 mM NaCl); Blue curve represents PRA spectra of AuNPs with ATP and Zr(IV) (30 mM NaCl). *c*(ATP): 10.0 μM; *c*(Zr(IV)): 10.0 μM.

Figure 1 shows that the PRA spectrum of the as-prepared AuNPs has a characteristic peak at 520 nm, indicating that AuNPs are well-dispersed in no presence of 30 mM NaCl. Under the fixed ionic strength of 30 mM, AuNPs are still in the dispersed state after the addition of 10 μM ATP, which can be identified by the characteristic peak at 520 nm. It suggests that ATP can adsorb onto the surface of AuNPs and keep AuNPs in the dispersed state. However, as shown in Figure 1, the absorption peak at 520 nm decreases and a higher absorption peak at 700 nm emerges in the presence of Zr(IV) (10 μM). It indicates that AuNPs get aggregated in the presence of Zr(IV) (10 μM). In the presence of both Zr(IV) (10 μM) and ATP (10 μM), the absorption peak at 520 nm is still high and only a much lower absorption peak at 650 nm emerges. It suggests that ATP can effectively prevent Zr(IV)-induced aggregation of AuNPs. 

### 3.2. Kinetic Behavior of PRA Sensor for ATP

**Figure 2:**
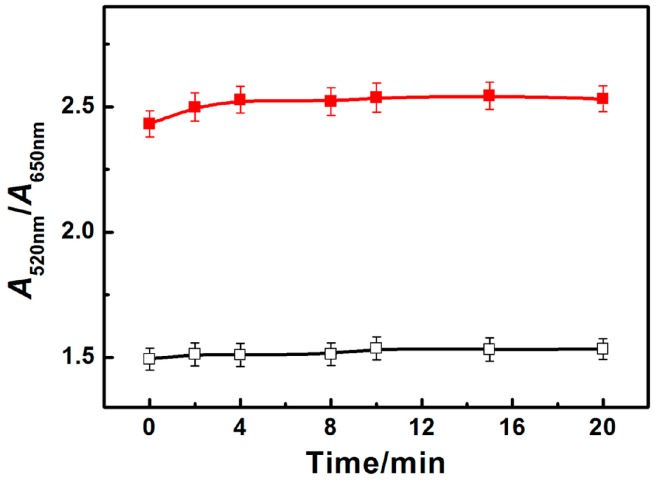
Kinetic behaviors of PRA sensor for ATP. Time (min): immediately, 2, 4, 8, 10, 15, 20; *c*(ATP, μM): 10; *c*(Zr(IV)): 10.0 μM. Black curve represents PRA spectra of AuNPs with Zr(IV); Red curve represents PRA spectra of AuNPs with Zr(IV) and ATP.

Figure 2 shows the kinetic behavior of the PRA sensor for ATP. After the addition of Zr(IV), AuNPs get aggregated immediately. The *A*_520nm_/*A*_650nm_ values in the PRA spectra of AuNPsin the presence of Zr(IV) keep stable during a period of 20 min. To achieve the proposed sensor for ATP, ATP is first added to react with Zr(IV) to form phosphate and Zr(IV) (–PO_3_^2−^–Zr^4+^–), and then AuNPs are added. Zr(IV)-induced aggregation of AuNPs is effectively prevented, enabling the detection of ATP. The *A*_520nm_/*A*_650nm_ values of AuNPs keep at stable and high values from the beginning (within 2 min) to 20 min. PRA results indicate that Zr(IV)-induced aggregation of AuNPs is stable and the detection of ATP is a fast process. The fast response is attributed to the fast interaction of phosphate and Zr(IV) [37,39].The fast response is favorable forreal-time or on-site detection of ATP. To achieve reliable and stable PRA signals for ATP detection, the optimized detection time is kept at 2 min.

### 3.3. Visual Sensor for ATP

**Figure 3:**
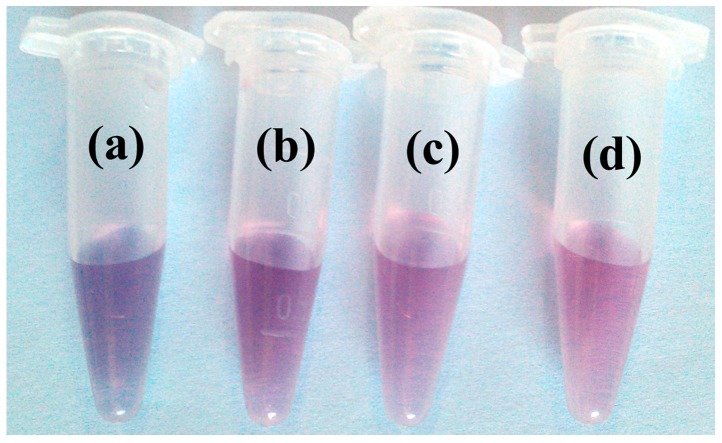
Photographsof ATP detection in the presence of Zr(IV). *c*(ATP, μM) from sample (**a**–**d**): 0, 0.5, 5.0, 10.0; *c*(Zr(IV)): 10.0 μM.

**Figure 4:**
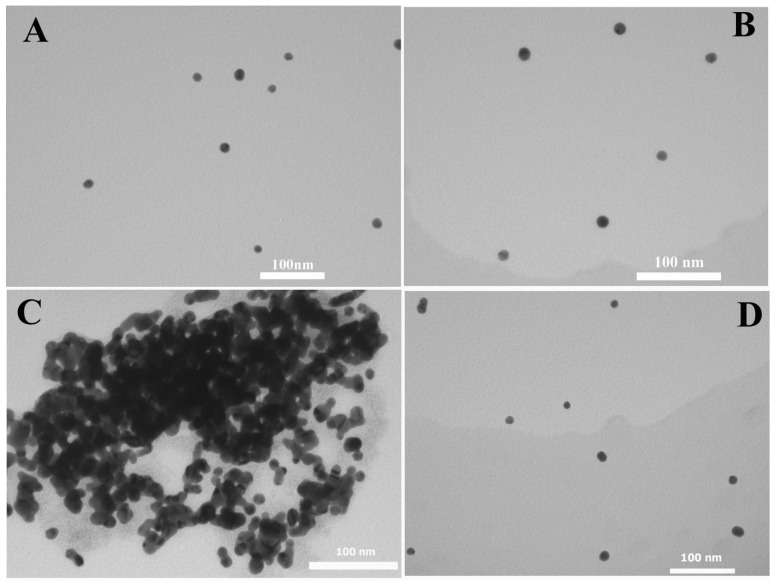
TEMimages of AuNPs alone (**A**); AuNPs with ATP (**B**); AuNPs with Zr(IV) (**C**); and AuNPs with Zr(IV) and ATP (**D**). *c*(Zr(IV)): 10.0 μM; *c*(ATP): 10.0 μM.

Due to the unique size-dependent optical properties of AuNPs, the color of dispersed AuNPs is usually red and while the color of the aggregated AuNPs is purple and even blue, which depends on the size of the aggregated AuNPs. As shown in Figure 3, the concentrations of ATP change from 0.5 to 10.0 μM in the fixed Zr(IV) (10 μM) condition;the color changes of the AuNP system from blue to red happen at the same time. Though the limit of detection (LOD) of 0.5 μM appears relatively high, it is in accordance with that of normal colorimetric or visual detections [32,34,35,36,42]. Since visual detections have advantages of simplicity and fast response, they are mostly applied in fast on-site detection. More accurate detection results can be obtained by sophisticated equipment (such as PRA detection as mentioned below). The aggregated and dispersed state is also confirmed by TEM measurements. As shown in Figure 4, AuNPs alone are dispersed (Figure 4A). After the addition of ATP, AuNPs are still dispersed in the AuNP and ATP system (Figure 4B). However, AuNPs get greatly aggregated in the AuNP and Zr(IV) system (Figure 4C). AuNPs are dispersed in the AuNPs, ATP and Zr(IV)ATP system (Figure 4D). Herein, both TEM and color changes confirm the feasibility of the proposed visual sensor for ATP using AuNPs. 

We also tested the mechanism of visual detection by changing the adding orders. By first adding ATP to AuNPs to enable ATP-AuNP binding, AuNPs get dispersed, and then when adding the Zr(IV), AuNPs get aggregated. It is the adding order used in our previous results for Zr(IV) detection ([41]). By first adding Zr(IV) to AuNPs to enable aggregation, then adding ATP to sequester Zr(IV), AuNPs can get dispersed. However, the extent of dispersed is not better than that achieved by first adding Zr(IV) and ATP and then adding AuNPs in the present work. To achieve the great color changes of AuNPs (the maximum background: signal ratio for ATP detection), we achieve the well-dispersed of AuNPs by first adding Zr(IV) and ATP and then adding AuNPs. 

### 3.4. Characterization of PRA Sensor for ATP

**Figure 5:**
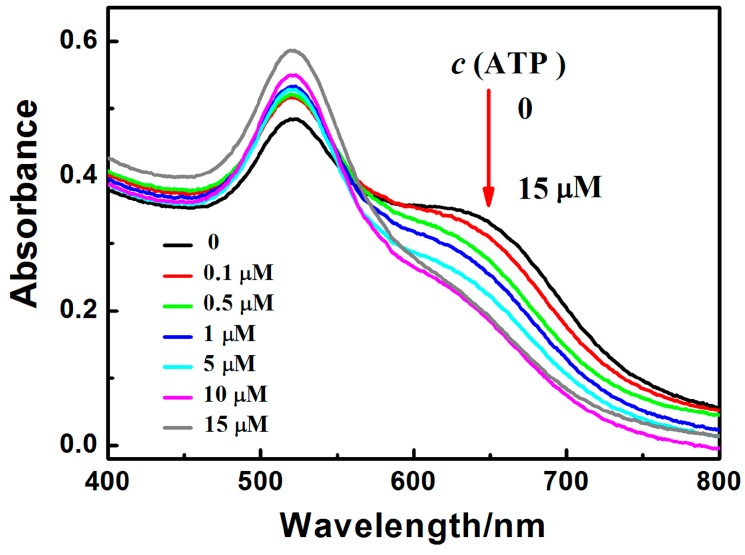
Absorption spectral results for ATP detection in the presence of Zr(IV). *c*(ATP, μM): 0, 0.1, 0.5, 1, 5, 10, 15; *c*(Zr(IV)): 10.0 μM.

**Figure 6:**
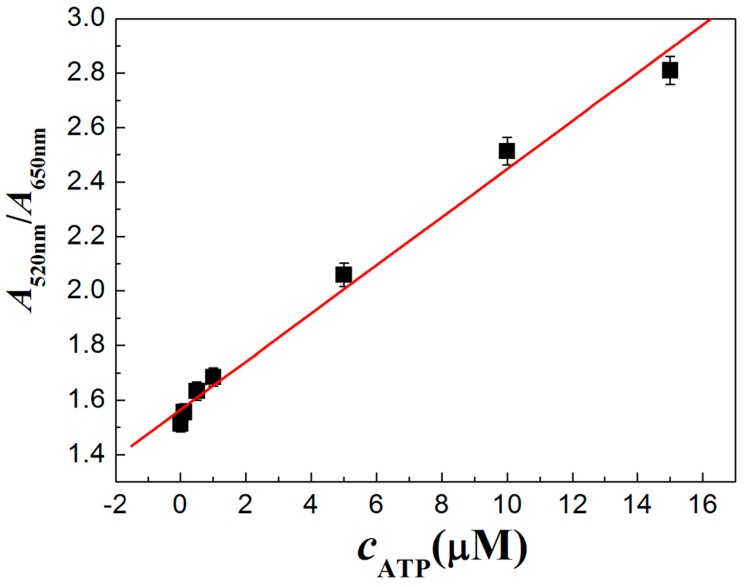
Linear relationship of ATP detection in the presence of Zr(IV). *c*(ATP, curve 1 to 7, μM): 0, 0.1, 0.5, 1, 5, 10, 15; *c*(Zr(IV)): 10.0 μM. The error bars represent the standard deviation of three measurements.

Figure 5 shows absorption spectral results at different concentrations of ATP. With the increasing concentrations of ATP, the absorbance at 650 nm decreases and the absorbance at 520 nm increases. It indicates that ATP induces AuNPs and the Zr(IV) mixture changes gradually from the aggregated state to the dispersed state. From the statistics of the absorption peaks in Figure 6, it can be seen that the values of *A*_520nm_/*A*_650nm_ increase linearly with the concentrations of ATP from 0.1 μM to 15 μM (r = 0.9945). The LOD is 28 nM, which is much lower than that of most other reports (Table 1). The LOD is calculated on the basis of the standard deviation (SD) of the blank measurements and the slope of the calibration curve (S) (limit of detection = 3SD/S) [41,43]. The LOD is comparable to some reports; the proposed sensor for ATP using AuNPs is simple and fast within 2 min. More importantly, the proposed sensor only needs the participation of inorganic ions of Zr(IV) without the need for aptamers, which saves the incubating time of ATP with its aptamer and cost. Therefore, the proposed sensor is available for real-time or on-site detection of ATP.

**Figure 7:**
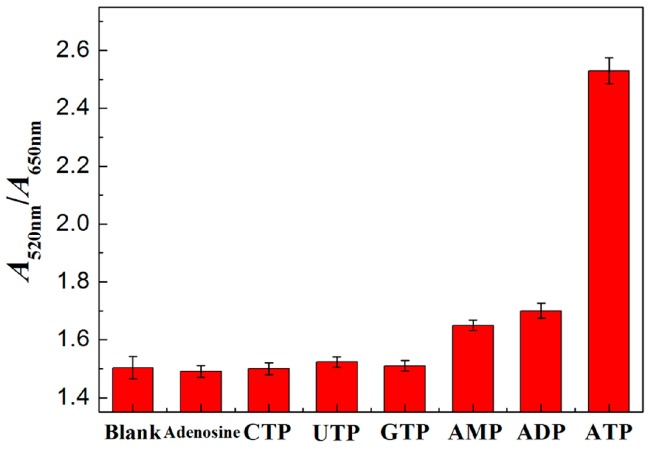
Selectivity for ATP detection. *c*(ATP and its analogs): 10 μM; *c*(Zr(IV)): 10.0 μM. The error bars represent the standard deviation of three measurements.

The selectivity of the proposed sensor for ATP is investigated in Figure 7. Compared with the values of the control sample (blank sample, nearly 1.5), the values of *A*_520nm_/*A*_650nm_ of other analogues such as adenosine, GTP, CTP and UTP are nearly 1.5 and the values of *A*_520nm_/*A*_650nm_ of AMP are nearly 1.65. However, the value of *A*_520nm_/*A*_650nm_ of ATP is high (nearly 2.5). According to some analytical results [44,45,46], a change of ±10% is regarded as a negligible error range. It can be seen that the proposed method for ATP detection has good selectivity over adenosine, GTP, CTP, UTP and AMP within ±10% error. The high selectivity is speculated as being due to three phosphate groups of ATP bringing much stronger affinity between Zr(IV)with ATP than adenosine or the other two phosphate groups (ADP) or one phosphate group’s derivatives (AMP). ATP can form a very stable phosphate and Zr(IV) (–PO_3_^2−^–Zr^4+^–) with Zr(IV). Besides, the high adsorption of adenosine onto the surface of the AuNPs leads to high selectivity for ATP over GTP, CTP and UTP [32,47]. The value of A_520nm_/A_650nm_ of ADP is nearly 1.70 beyond the ±10% error. It may be attributed to the two phosphates of ADP having a higher affinity for Zr(IV) than the one phosphate of AMP, but they have a weaker higher affinity than the three phosphates of ATP. It indicates the higher affinity of ADP with Zr(IV) than AMP with Zr(IV).

### 3.5. Detection of ATP in Synthetic Mixture

Usually the amount of ATP in cells or bacterium is from 10^−18^ to 10^−15^ mol per cell [49]. Therefore, most reports detect ATP in a real urine sample or diluted serum sample by adding a higher concentration of ATP standard solution. In commercial ATP injections (20 mg/2 mL), it can be achieved by our present method for ATP detection. To investigate the feasibility of the proposed sensor to measure real samples, a synthetic mixture containing adenosine, ADP, AMP, GTP, CTP and UTP, as mentioned above, are utilized in recovery measurements. ATP (1, 5 and 10 μM) was respectively added to synthetic mixtures (Table 2). Then Zr(IV) (10 μM) and tris-HCl buffer solutions (pH 7.4, 100 μL) were added to the above solutions to achieve the affinity between Zr(IV) and ATP. Finally, AuNPs and NaCl (30 mM) were added. The whole solutions were vortex-mixed thoroughly, kept at room temperature for 2 min and transferred for PRA spectrum measurements. Since the synthetic mixtures did not have ATP, recovery percentages are calculated by dividing the concentrations of ATP measured with the added concentration of ATP. The calculated mean recoveries for the analysis of ATP range from 95.3% to 102.0% by three measurements. The satisfactory results indicate that the proposed PRA and visual sensor is promising in the application of real samples.

## 4. Conclusions

In conclusion, a new visual and PRA sensor for ATP has been developed using AuNPs based on the high affinity between Zr(IV) and phosphate. It achieves visual detection of ATP with a LOD of 0.5 μM within 2 min. It realizes PRA detection for ATP from 0.1 μM to 15 μM with a LOD of 28 nM, which is comparable to other reports. The proposed sensor for ATP using AuNPs is simple and with a fast response within 2 min. More importantly, the proposed ATP sensor only needs the participation of inorganic ions Zr(IV) free from aptamers, which saves the incubating time of ATP with its aptamer. The proposed sensor exhibits good selectivity for ATP over other analogs such as adenosine, AMP, GTP, CTP and UTP. In addition, the recoveries for the analysis of ATP in synthetic samples are satisfactory. Therefore, the proposed ATP sensor can potentially be applied in real-time or on-site detection of ATP.

## Figures and Tables

**Table 1:** Comparison of different methods for ATP detection.

Methods	Materials	Linear Range (μM)	LOD (nM)	Refs.
ECL(using aptamer)	CdSe/ZnSquantum dots	0.018~90.7	6	[13]
ECL(using aptamer)	Magnetic nanoparticles-CdSe/CdS quantum dots	0.01~0.8	3	[12]
ECL(using aptamer)	[Ru(bpy)_3_]^2+^, single-walled carbonnanohorn	0.005~50	1	[9]
ECL(using aptamer)	[Ru(bpy)_2_dppz]^2+^	0.2~1	100	[10]
FRET(using aptamer)	FAM-labelled DNA, graphene oxide	3~320	450	[20]
FRET(using aptamer)	FAM using SDR amplification	0.02~0.6	20	[21]
FRET(no aptamer)	The prepared ratiometric fluorescent probe: naphthalimide-rhodaminecompound	0.1~10	100	[19]
Fluorescence(using aptamer)	SYBR Green I using exonuclease-catalyzed target recycling amplification	0.01~2	9.5	[22]
LSPR(using aptamer)	Gold nanorod, TAMRA dye	0.00001~10	10 pM	[1]
ITC(using aptamer)	Glucose oxidase	10~100	10 μM	[48]
DPV(using aptamer)	Porphyrin functionalized graphene nanosheets	0.0022~1.3	0.7	[15]
Amperometry(no aptamer)	Glucose oxidase and hexokinase co-immobilizedPt electrode	100~16000	2500	[16]
PRA(no aptamer)	AuNPs, Zr(IV)	0.1~15	28	This work

SWV: square-wave voltammetry; ECL: electrochemiluminescence; FRET: fluorescence resonance energy transfer; FAM: carboxyfluorescein; SDR: strand displacement reaction; LSPR: localized surface plasmon resonance; ITC: isothermal titration calorimetry; DPV: differential pulse voltammetry.

**Table 2:** Recoveries for ATP detection in synthetic samples.

Samples	The Added ATP (μM)	The Total ATP (μM)	Mean Recoveries (%)
1	1	0.98, 1.03, 1.05	102.0
2	5	5.04, 5.01, 5.11	101.1
3	10	9.87, 9.93, 10.05	95.3
